# Increased Vigilance Needed for the Detection of Thrombotic Complications of Central Venous Access in Adolescent Cystic Fibrosis Patients

**DOI:** 10.3389/fped.2014.00117

**Published:** 2014-11-19

**Authors:** Nandini Kandamany, Basil Elnazir, Peter Greally

**Affiliations:** ^1^Paediatric Cystic Fibrosis Department, Adelaide and Meath National Children’s Hospital, Tallaght, Dublin, Ireland

**Keywords:** cystic fibrosis, thrombotic, thromboembolic, totally implantable venous access device, Port-A-Cath, indwelling venous catheter

## Introduction

Cystic fibrosis (CT) is a genetically inherited, multisystem condition, resulting from abnormal salt and water transport across epithelial surfaces. Advances in medical treatments have resulted in vastly improved care of CF patients. This has led to the increased survival of CF sufferers from early childhood in the 1940s to nearly 40 years of age in 2012 (1). These therapies include nebulized and inhaled mucolytics, and bronchodilators, as well as prophylactic antibiotics and physiotherapy. Most CF patients require some form of long-term venous access for ease of administration of repeated courses of intravenous antibiotics. Some of our patients are fitted with a totally implantable venous access device (TIVAD) referred to as a Port-A-Cath. Over time, these indwelling devices present their own complications. We report a series of three patients with potentially life-threatening thrombotic and thromboembolic phenomena arising from their indwelling central venous catheters (CVCs).

## Case 1

A 16-year-old Caucasian female CT (ΔF508/ΔF508) patient with pancreatic insufficiency and CF-related diabetes mellitus presented to our unit 2 years post-right-sided chest Port-A-Cath (indwelling CVC) insertion. She had developed acute hemoptysis and epistaxis with associated pleuritic chest pains. On auscultation of her chest, air entry was equal with no localized crepitations. Her pulmonary function tests revealed an FEV1 of 0.58 (23%) and an FEF of 25–75.17 (7%). These measurements were reasonably close to her baseline. A chest X-Ray revealed no evidence of pneumothorax. Her full blood count and coagulation screen were normal. Her symptoms resolved spontaneously and she was discharged.

**Figure 1:**
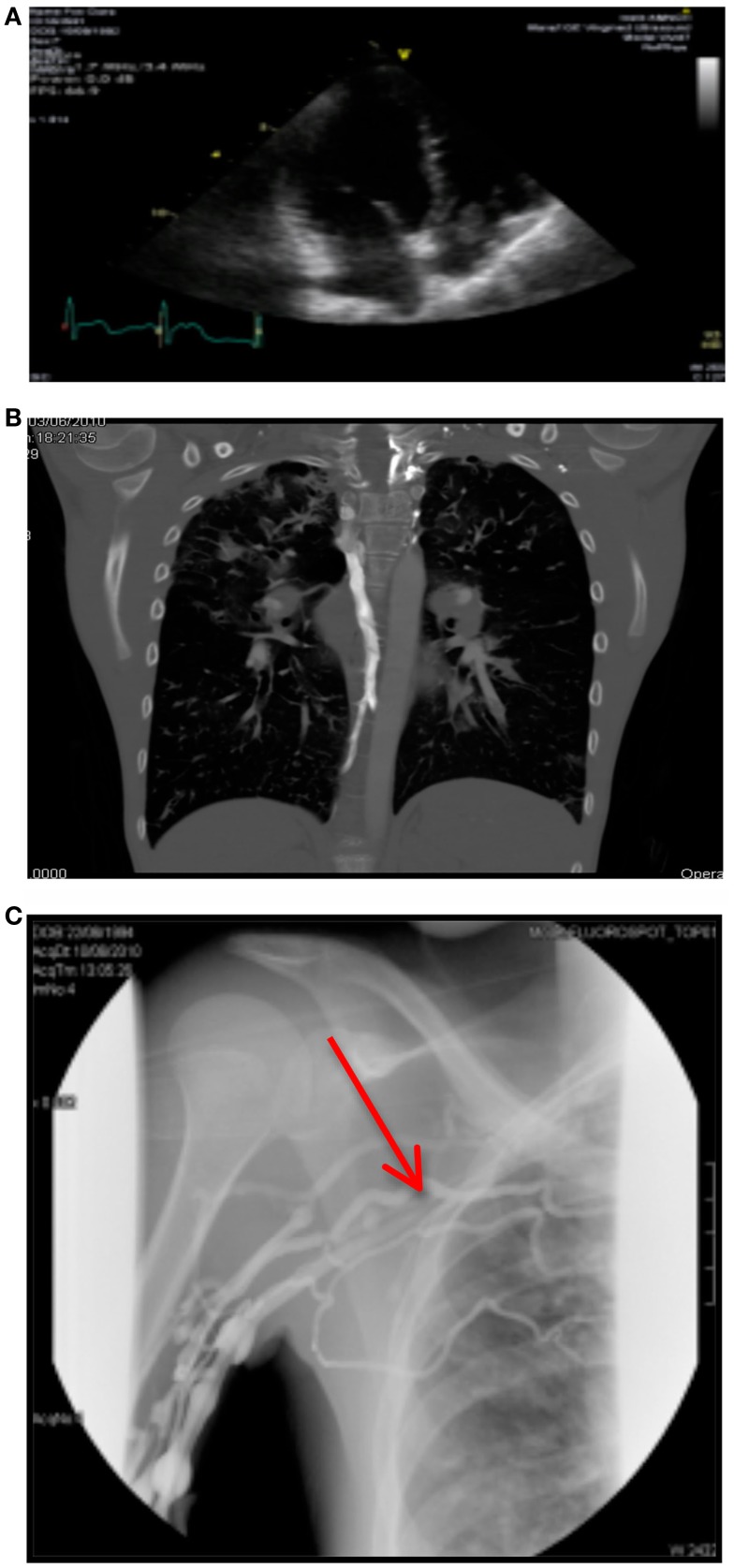
**(A)** Right atrial thrombus on echocardiography. **(B)** CTPA showing bilateral arterial filing defects. **(C)** Venogram showing complete obstruction of right subclavian vein.

A month later, her Port-A-Cath developed complete resistance on flushing during routine home intravenous antibiotics. A venogram showed a small thrombus at the tip of her Port-A-Cath, causing no obstruction. The patient was asymptomatic. An echocardiogram was performed 4 days later and revealed a very large, well-organized *right atrial thrombus* at the level of the tricuspid valve, attached to the Port-A-Cath tip (see Figure 1A). The right ventricle appeared normal.

Her coagulation screen was repeated and remained normal. However, her Anti-Factor Xa level was low 0.02. Thrombolytic therapy was initiated with Alteplase 2 mg instilled into her venous catheter for 4-h periods and she was commenced on a 4-week course of tinzaparin 175 units/kg/day.

## Case 2

An 18-year-old Caucasian male CF patient (ΔF508/G551D) with pancreatic insufficiency, CF-related liver disease, and impaired glucose tolerance had an indwelling CVC inserted into his right internal jugular vein. This was his eighth catheter in 7 years. A week post-insertion, it was noted to be difficult to draw blood from this catheter. A venogram was subsequently performed, which showed a *right internal jugular venous thrombus*, with very poor flow through his right subclavian and external jugular veins. The catheter was removed and a right femoral venous catheter was inserted under radiological guidance with difficulty due to poor venous flow. He was treated with anti-coagulants.

Less than a week later, he developed acute dyspnea, and a progressively increasing oxygen requirement. Examination of his chest revealed scattered crepitations bilaterally with no focal crepitations detected. His femoral venous catheter was no longer functioning, leaving the patient with no venous access.

On pulmonary function testing, his FEV1 was 16% and his FVC was 31%, both slightly below baseline. The rest of his routine bloods including coagulation screen were normal. A chest X-Ray was performed, which showed no pneumothorax. A CT pulmonary angiogram (CTPA) was scheduled, but was unable to be performed due to the complete lack of patent venous access. A VQ scan was considered for *suspected pulmonary emboli*. However, upon discussion, this was also deemed impractical due to the patient’s lack of venous access. He was commenced on therapeutic enoxaparin sodium (Clexane) at 1 mg/kg/bd SC, which was monitored with anti-Factor Xa levels.

## Case 3

A 15-year-old Caucasian male with G551D/n CT, pancreatic insufficiency and a PEG *in situ* presented to hospital with dyspnea, a non-productive cough and reduced exercise tolerance. His oxygen saturations were 88% in room air. There were no focal crepitations detected and air entry was equal bilaterally. His routine blood results were all within the normal ranges including a coagulation screen. No pneumothorax was noted on chest X-Ray. His chest Port-A-Cath appeared to be functioning well. He was admitted and intravenous antibiotics were commenced. A high-resolution CT scan showed chronic CT changes and right-sided volume loss.

Subsequently, a CTPA was performed. This revealed bilateral arterial filling defects with a prominent azygos vein, indicating elevated right atrial pressure (see Figure 1B).

His venogram showed normal central line flow with *complete obstruction of the right subclavian vein and multiple collateral filling defects* (see Figure 1C).

His D-dimers 3 days post-admission were elevated to 1.26. He was commenced on warfarin 5 mg/kg, low-molecular weight heparin (LMWH) × 5/7, and low-dose ADEK for right subclavian vein thrombosis and possible pulmonary emboli.

## Discussion

Intravenous antibiotics are the mainstay of treatment for infective exacerbations of CF. However, intravenous cannulation is recognized as a fear-inducing and often traumatic process for children, particularly as repeated attempts at cannulation are often necessary in patients with a chronic illness. It follows that obtaining venous access by the least traumatic and most effective means is an important aspect of the management of children who require repeated courses of intravenous antibiotics. TIVADs were originally utilized in the treatment of oncology patients. The use of TIVADs in children with CF was first described in 1986 (2). These devices require minimal care, do not limit physical activity and are relatively tamper-proof (3). In addition, they are cosmetically acceptable and allow ease of access to large, central veins, which are more likely to withstand repeated courses of intravenous treatment.

Thrombotic and thromboembolic complications of TIVADs have been widely reported. These range from microthrombi occluding the tip of venous catheters to potentially life-threatening venous thrombosis (VTs), occasionally, resulting in pulmonary and paradoxical emboli. In 1997, Deerojanawong et al. reported a symptomatic VT rate of 9% in CF patients in Melbourne, VIC, Australia (3). Munck et al. indicated a thrombosis rate of 4.7% in 2004 (4) and in 2008, a retrospective review over 20 years of a single center in Birmingham, England reported 5 cases of VT in 105 patients (165 TIVADs) (5). Several case reports of paradoxical embolization have been reported in adult CF patients (6–8) and one in an 11-year-old CF sufferer (9). The placement of TIVADs itself is felt to induce a pro-thrombotic response through local inflammation and the presence of foreign material intravascularly. In addition to these acquired mechanical risk factors, VT in CF patients is felt to be in part due to underlying abnormalities of coagulation.

The tendency toward hypercoagulability in CF patients has been a subject of much research. Takemoto et al. suggest that in addition to CVCs, acquired thrombophilia secondary to inflammation and deficiencies of anticoagulant proteins due to vitamin K or liver dysfunction are likely to contribute to the increased risk of thrombosis in CF (10). Furthermore, children with CF appear to have a higher frequency of protein C and S deficiencies than expected for inherited deficiencies (11). Deficiencies in both these proteins are possibly due to vitamin K deficiency or hepatic dysfunction (12). Balfour-Lynn et al. also reported greater than expected rates of protein C deficiency (4 vs. 0.2–0.4%) and protein S deficiency (5 vs. 0.3%) in addition to anti-thrombin deficiency (1 vs. 0.2%) and lupus anticoagulant deficiency (9 vs. 1–5%) in CF patients compared to the general population (13). This study of 204 CF patients was conducted in asymptomatic patients in an out-patient setting, and thus, did not investigate levels in patients with confirmed VT. Another study of 71 patients concurred with Balfour-Lynn et al. reporting elevated rates of lupus anti-coagulants (18%), protein C deficiency (14%), and protein S deficiency (19.7%) (14). Anti-phospholipid antibody levels have also been noted to be raised in children with CF (4–9.5 vs. 1–3%) (15, 16). In addition, VT has been reported at a rate of 27% in those with Burkholderia Cepacia colonization, though the exact mechanism of this relationship has not been ascertained (12).

Symptoms of thrombotic complications in CF patients may mimic those of infective pulmonary exacerbations, thus, causing a delay in diagnosis. Two of the patients in our case series presented with an increased oxygen requirement and progressively worsening dyspnea, both common symptoms of lower respiratory tract infections in CF patients. The other patient in our case series presented with hemoptysis, also a symptom seen frequently in CF patients, due to chronic lung inflammation. One of our patients had a normally functioning indwelling CVC, but was only noted on venogram to have developed a complete obstruction of his right subclavian vein. This suggests that the ease of blood flow from an indwelling catheter is not a reliable gage of venous patency due to the potential for the development of collaterals.

While TIVADs are not the sole risk factor for VTs in CF patients, the risk of venous thromboembolism (VTE) does increase with the duration of the CVCs (4). Following a multicentre, prospective cohort study of 80 patients, Dal Molin et al. reported a mean of 203.6 days between TIVAD positioning and the onset of thrombosis (17). The age of first diagnosis of CF is decreasing and given the recent introduction of newborn screening for CF in our patient population, it follows that patients will be fitted with their first TIVAD at an earlier age, which increases the chances of long-term complications by the time they reach adolescence. Therefore, it is our opinion that there is a need for more stringent screening for thrombotic and thromboembolic complications from an earlier age.

In a recent prospective, multicenter, observational study assessing central VTs and thrombophilia in CF, Munck et al. demonstrated a 50% hypercoagulability rate and a 6.6% catheter-related VT rate. Interestingly, patients who had previously had a central line were noted to have a 9.1% rate of catheter-related VT on Doppler ultrasound (prior to TIVAD insertion). Five out of six patients were asymptomatic at the time of diagnosis of VT using Doppler ultrasound; 4 prior to TIVAD insertion, 1 at 1-month follow-up scan, and 1 at 6-month follow-up scan. Munck et al. concluded that laboratory thrombophilia screening was poorly predictive and thus not recommended routinely in patients prior to TIVAD insertion, but that a focused medical history (assessing for thrombotic risk factors) and prospective Doppler US scans might be useful in identifying asymptomatic catheter VT (18).

Given the evidence we have reviewed, we suggest that an extended coagulation panel (incorporating at a minimum, protein C, protein S, anti-thrombin, and factor VIII and factor V Leiden levels) and Doppler ultrasound screening be conducted at regular intervals in patients with TIVADs who have had a history of a VT, as well as prior to TIVAD insertion. Patients at risk for VT (family history of hypercoagulability, oral contraception, etc.) will need close surveillance and the role of Doppler ultrasound should be considered in these cases.

Several studies researching the benefit of thromboprophylaxis in CF patients have been conducted. In 1992, Sola et al. recommended the prophylactic use of aspirin in all CF patients without evidence of liver disease or coagulopathy (19). There has been no efficacy shown for prophylaxis with low-dose warfarin therapy (INR 1.3–1.9) to prevent CVC-associated thrombosis. Of particular, concern is the risk of hemoptysis, which is associated with CF (20).

All three of our patients developed their thrombotic complications despite the use of recommended flushing using the “stop and go” technique. While local guidelines prevail at each CF center, it is accepted that local CVC obstructions secondary to fibrin sheaths may be managed with infusions of thrombolytics such a tPA in the first instance, and do not warrant systemic anticoagulation (10). Acute, symptomatic DVT associated with central lines should be managed initially with heparin therapy then removed after 3–5 days or until a therapeutic level of anticoagulation is achieved to reduce the risk of embolization (21) The optimum duration of treatment has not been extensively studied, but Munck et al. recommended 3–6 months of anticoagulation for patients with CVC-related thrombosis (4). There has also been a move toward using peripherally inserted central catheters (PICCs) in CF patients, which are not without their own complications.

In conclusion, the marked improvement in the early detection and survival of CF patients has seen a concomitant rise in challenges faced by late childhood and early adolescence in this group of patients. We believe our case series highlights an important phenomenon that is likely to feature more prominently as the average age of CF survivors increases. Presently, there is no clear consensus as to whether to routinely screen CF patients to identify those at risk for significant thrombosis prior to the development of VT. Our recommendation that those who have a history of a previous VT and those with a significant family history of this should potentially undergo an extended thrombophilia screen is based on the best available evidence. The use of directed Doppler ultrasound prior to TIVAD insertion, and at fixed intervals thereafter, may help identify the formation of catheter-related VT prospectively. Thromboprophylaxis cannot be recommended routinely at this stage. We strongly feel this is an area that would benefit from larger and more robust studies to achieve early identification of CF patients who are at risk for serious thrombotic and thromboembolic complications arising from their indwelling central lines.

## Conflict of Interest Statement

The authors declare that the research was conducted in the absence of any commercial or financial relationships that could be construed as a potential conflict of interest.
